# Leveraging AI to improve disease screening among American Indians: insights from the Strong Heart Study

**DOI:** 10.3389/ebm.2024.10341

**Published:** 2025-01-08

**Authors:** Paul Rogers, Thomas McCall, Ying Zhang, Jessica Reese, Dong Wang, Weida Tong

**Affiliations:** ^1^ National Center for Toxicological Research, Division of Bioinformatics and Biostatistics, U.S. Food and Drug Administration, Jefferson, AR, United States; ^2^ Department of Data Science and Data Analytics, Arkansas State University, Jonesboro, AR, United States; ^3^ University of Oklahoma Health Sciences Center, Department of Biostatistics and Epidemiology, Oklahoma City, OK, United States

**Keywords:** artificial intelligence, machine learning, screening test, American Indian, low prevalence

## Abstract

Screening tests for disease have their performance measured through sensitivity and specificity, which inform how well the test can discriminate between those with and without the condition. Typically, high values for sensitivity and specificity are desired. These two measures of performance are unaffected by the outcome prevalence of the disease in the population. Research projects into the health of the American Indian frequently develop Machine learning algorithms as predictors of conditions in this population. In essence, these models serve as *in silico* screening tests for disease. A screening test’s sensitivity and specificity values, typically determined during the development of the test, inform on the performance at the population level and are not affected by the prevalence of disease. A screening test’s positive predictive value (PPV) is susceptible to the prevalence of the outcome. As the number of artificial intelligence and machine learning models flourish to predict disease outcomes, it is crucial to understand if the PPV values for these *in silico* methods suffer as traditional screening tests in a low prevalence outcome environment. The Strong Heart Study (SHS) is an epidemiological study of the American Indian and has been utilized in predictive models for health outcomes. We used data from the SHS focusing on the samples taken during Phases V and VI. Logistic Regression, Artificial Neural Network, and Random Forest were utilized as *in silico* screening tests within the SHS group. Their sensitivity, specificity, and PPV performance were assessed with health outcomes of varying prevalence within the SHS subjects. Although sensitivity and specificity remained high in these *in silico* screening tests, the PPVs’ values declined as the outcome’s prevalence became rare. Machine learning models used as *in silico* screening tests are subject to the same drawbacks as traditional screening tests when the outcome to be predicted is of low prevalence.

## Impact statement

Artificial Intelligence (AI) and Machine Learning (ML) techniques are increasingly integrated into screening and diagnostic models to pinpoint individuals at risk of specific diseases or medical conditions. However, with the rise in popularity of AI and ML, the literature (and internet) is flooded with reports on computer-based prediction and screening tests, often focusing more on showcasing the technique rather than discussing their screening and diagnostic performance. In particular, there is a proliferation of algorithms created for minority groups, including the American Indian. A motivating factor in creating an *in silico* screening exam for American Indians is that this population, as a whole, experiences a greater burden of comorbidities, including diabetes mellitus, obesity, cancer, cardiovascular disease, and other chronic health conditions, than the rest of the U.S. population. This report evaluates these AI algorithms for the American Indian like a screening test in terms of performance in low prevalence situations.

## Introduction

Artificial Intelligence (AI) and Machine Learning (ML) techniques are increasingly integrated into screening and diagnostic models to pinpoint individuals at risk for specific diseases or medical conditions [1]. However, with AI’s and ML’s rise in popularity, the literature (and the internet) is flooded with reports on computer-based prediction and screening tests, often focused more on showcasing techniques than discussing their screening and diagnostic performance. Advances in computer processing speed, increasing numbers of data scientists, low- to no-cost programming libraries, and availability of larger healthcare data sets have driven the proliferation of AI algorithms [2]. Kumar et al. have listed a sampling of prediction algorithms and data sets, including those for outcomes in Alzheimer’s disease, cancer, diabetes, chronic heart disease, tuberculosis, stroke, hypertension, skin disease, and liver disease, among others [3]. Notwithstanding the proliferation of algorithms, AI is positioned to considerably enhance the accuracy and efficiency of screening tests. Specifically, ML algorithms can be trained on extensive data sets to discern patterns and make predictive analyses based on those patterns. Rapid expansion of AI technology, coupled with enhanced computing power in health screening, underscores the necessity for evaluating the algorithm’s performance and quality of these algorithms [4].

The U.S. Government Accountability Office conducted a technology assessment noting that AI and ML offer advantages in analyzing underserved populations [5]. However, one challenge of utilizing AI in epidemiology pertains to the underrepresentation or absence of minority groups within these algorithms’ training data sets [6]. Also, screening test performance may vary in minority populations due to their differences in disease prevalence from non-minority populations.

Many AI and ML methods for predicting disease in non-minority populations are recalibrated for minority groups. For example, an ML algorithm for mortality prediction based on chronic disease was recalibrated for the population of South Korea; this adjusted index showed a greater mortality prediction than the original algorithm [7]. Another effort adjusted this mortality prediction algorithm using hospital discharge abstracts from six countries [8].

In terms of minority status, American Indians are sometimes referred to as the “minority of the minority” or the “invisible minority,” given their small population, cultural identity, languages, and histories that set them apart from other groups. Focusing on AI and ML can offer advantages in analyzing these underserved populations, who, like American Indians, bear a greater burden of certain health conditions [9–11]. This study focused on *in silico* AI and ML screening tests explicitly designed for the American Indian population.

The number of *in silico* diagnostic and screening tests has grown exponentially over the last decade, with many of these utilizing data sets based on American Indians. Our study serves as a reminder that *in silico* screening tests, even when classified as AI or ML algorithms, are still subject to the same limitations related to disease prevalence as those of their laboratory-based counterparts.

### Popularization of Pima Indian data

Several research articles in the public domain report on AI and ML algorithms for diabetes classification in the Pima Indian population. A contributing factor is the availability of numerous Pima Indian data sets provided to the AI community through platforms like Kaggle, a popular resource for AI and ML algorithm developers [12]. However, these studies often overlooked the differences in disease prevalence among different populations and the potential consequences of applying algorithms trained specifically on one population to another.

Examples of ML algorithms for diabetes classification in Pima Indians sampled from the literature include Support Vector Machines, Radial Basis Function, Kernel Support Vector Machines, K-Nearest Neighbor, Artificial Neural Networks, Fuzzy Support Vector Machine, Naïve Bayes Classifier, J48 Decision Tree, and a Random Forest Classifier [13–15]. Some of the articles in this sample failed to recognize the high prevalence of diabetes among the Pima Indians and the impact of disease prevalence on screening test performance, and tended to focus solely on the methods used to perform the classifications [14].

### The Strong Heart Study models

The Strong Heart Study (SHS) has played a significant role in identifying risk factors and patterns related to cardiovascular disease (CVD) in American Indian communities. It included 12 tribes located in Oklahoma, Arizona and the Dakotas. Statistical models developed using SHS data have informed interventions and public health policies targeting CVD. SHS data were also used in developing ML models and risk-based calculators addressing hypertension, diabetes, and coronary heart disease (CHD) [16–18].

### AI and ML risks with American Indian data sets

However, potential risks are also associated with using ML in American Indian contexts. One notable concern involves the risk of ML algorithms perpetuating biases and stereotypes about American Indian communities. Specifically, algorithms trained on data sets that reinforce biases and stereotypes about American Indians could inadvertently foster further inequities against a population group frequently underrepresented in AI/ML training data sets for applications such as virtual screening tests. A lack of training data can also result in inaccuracies; a recent example involved an image recognition application identifying an American Indian in native dress as a bird [19]. To mitigate such risks, ML researchers and developers need to collaborate closely with American Indian communities to ensure their technologies are developed ethically and respectfully. Collaborations could entail establishing research partnerships with American Indian communities, involving community members in designing and developing ML models, and ensuring that models are built using unbiased and culturally sensitive data sets, like those of the SHS.

### Traditional screening test performance

**FIGURE 1:**
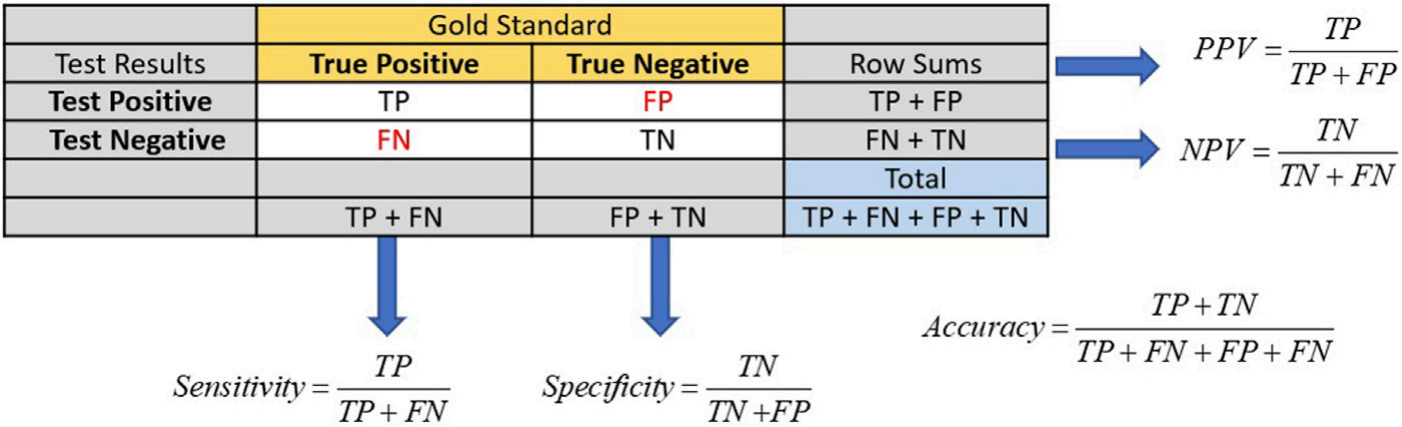
Calculations of sensitivity, specificity, PPV, and NPV for screening tests usually have their performance metrics determined via a gold standard. The numbers of true positives and negatives are represented by TP and TN, respectively. Likewise, the numbers of false positives and negatives are represented by FP and FN.

Traditional screening test performance is typically based on a gold standard in which an individual’s true disease status is known to establish the test’s sensitivity, specificity, positive predictive value (PPV), and negative predictive value (NPV). The test’s sensitivity and specificity inform its effectiveness in identifying the proportion of people in the population with and without the condition of interest [20]. Sensitivity is the ability of the test to correctly identify those with the condition, while specificity is the ability of the test to correctly identify those without it. Sensitivity can be calculated from the column of those truly positive for the condition, while specificity is derived from the column of those truly negative for the condition in Figure 1.

Among these metrics, the PPV holds clinical significance for both healthcare providers and patients. The PPV is a conditional probability that the tested individual has the disease, given that they tested positive. A high PPV indicates effective identification of individuals with the tested condition, guiding further testing, diagnosis, and treatment decisions. The PPV is calculated from the row in Figure 1 that represents those subjects who tested positive for disease.

As disease prevalence decreases, screening test performance decreases, particularly concerning the PPV. This decline can lead to situations where accuracy and sensitivity remain high, giving a false impression of a well-performing test due to the increased number of false positives (FP). For example, in a population of 1,000 people with a disease prevalence of 40%, a test with a sensitivity of 90% and specificity of 80% will produce a PPV of 75%. If the disease prevalence is lowered to 10% in this same population, the PPV drops to 33.33%; hence, the prevalence dominates in screening for rare diseases [21]. Therefore, healthcare providers should consider these factors when interpreting the PPV for further testing and treatment decisions.

While sensitivity and specificity provide information about test performance across populations, PPV is often more relevant in clinical practice. It helps physicians assess the likelihood of disease presence after a positive test result, especially in populations with low disease prevalence. If the disease outcome becomes increasingly rare, the algorithm will likely always predict the absence of disease, leading to high accuracy but poor PPV [22].

This study aimed to develop and evaluate three popular and commonly used AI and ML techniques as *in silico* screening tools for predicting three chronic conditions with differing prevalences in the SHS population: peripheral artery disease (PAD), hypertension, and type 2 diabetes. Specifically, we predicted the disease outcome using epidemiological data with methods including artificial neural networks (ANNs), random forest (RF), and logistic regression (LR). Unlike their traditional laboratory-based counterparts, these *in silico* tests do not have pre-determined sensitivity or specificity; rigorous testing has not been performed using a gold standard to establish these values. Our simulations provided a glimpse of the sensitivity, specificity, and PPV of these *in silico* screening tests, as these values changed in response to differing disease prevalences. We hypothesized that these *in silico* screening tools tailored to the American Indian population would show reduced performance as disease prevalence declines, regardless of the AI or ML method.

This research serves as a reminder that the limitations of screening tests regarding disease prevalence still apply, whether those tests are *in silico* AI or ML algorithms or traditional screening tools.

## Materials and methods

LR, ANNs, and RFs are well-known methods for creating *in silico* screening tests. While RFs operate as a nonlinear model, LR requires a linear relationship with the regression coefficients. ANNs present a more intricate approach, often featuring multiple layers commonly known as deep learning. AI and ML can potentially enhance screening for various medical conditions by illuminating linear and nonlinear data relationships. Nonetheless, it is crucial to acknowledge that the application of AI in medical research and screening exams is still nascent, and concerns over AI algorithms’ accuracy and reliability linger.

### Longitudinal epidemiological SHS data set

The SHS began in 1988 as a multi-center, population-based longitudinal study of cardiovascular disease (CVD) and its risk factors among American Indians. The study had three phases: a clinical examination, a personal interview, and an ongoing mortality and morbidity survey [23]. Participants from 12 different American Indian tribes were recruited from Arizona, Oklahoma, and the Dakotas, aided by volunteers from each community who promoted participation [24].

Phase II of the SHS, examining changes in risk factors for CVD in the original cohort, occurred between 1993 and 1995. The Strong Heart Family Study (SHFS), launched in Phase III (1998–1999), investigated genetic determinants of cardiovascular disease and extended recruitment to the original cohort’s family members aged 18 years and older [25]. Phase IV (2001–2003) involved surveillance of the original cohort plus 90 families to continue the study of genetic markers for CVD [26]. The Phase V exam (2006–2009) continued the SHFS, which began in Phase III; all participants from Phase III and IV were invited to participate in examinations conducted at local Indian Health Service hospitals, clinics, or tribal community facilities [27]. In Phase VI (2014–2018), all surviving participants were invited to complete a medical questionnaire, and continued the morbidity and mortality surveillance continued.

Physicians on the SHS Morbidity and Mortality (M & M) review committee examined the types of health-related events requiring hospital treatment and subsequent causes of mortality, when it occurred. Two of these physicians independently reviewed fatal events for cause, with the results reconciled by a third physician. In addition, one physician reviewed the medical records regarding study participant’s non-fatal events to verify specific diagnoses (i.e., stroke). This surveillance occurred yearly for both the original cohort and family cohort participants.

The available 2,468 Phase V SHS participants were divided into development and training cohorts (80%), while the remaining sample (20%) was assigned to a testing cohort. The training cohort generated the model weights, while the testing cohort assessed the algorithm’s quality.

A 1-year time-to-event data set for this study was constructed from the examination date in Phase V. The M & M results and all Phases of SHS data will cumulatively provide information on the subject’s medical conditions and mortality outcomes. Basic descriptive demographic statistics by gender, age, and comorbidity, including the numbers and percentages for binary variables, are listed in Table 1.

**TABLE 1:** Age, gender, and medical condition of SHS Phase V participants.

Medical condition	All	Male	Female
N (%)	2,468	977 (39.59)	1,491 (60.41)
Age (years)			
Mean (SD)	45.55 (16.41)	43.74 (16.00)	46.73 (16.58)
Median	44.40	42.70	45.70
Hypertension (%)	948 (38.41)	402 (42.41)	546 (57.59)
Diabetes (%)	631 (25.56)	240 (38.03)	391 (61.97)
PAD (%)	94 (3.81)	32 (34.04)	62 (65.96)

Data labels for hypertension and diabetes already existed within the SHS Phase V data set but did not include a specific label for PAD. The data included the participants’ right and left ankle-brachial indexes (ABIs), which were used to define the presence or absence of PAD. This study used a resting ABI of less than 0.90 on either the right, left, or both sides, similar to that in Virane et al., to indicate a PAD diagnosis. Participants were coded as either 1 or 0 for the presence or absence of PAD, respectively [28, 29].

LR, ANN, and RF were then used to model the PAD, hypertension, and diabetes target features. These models ran 100 unique iterations of splitting and training the data, and producing metrics from the test set. Metrics tracked for the models were accuracy, specificity, sensitivity, PPV, and NPV, which were averaged over 100 iterations for each model type.

SAS version 9.4 was used to assemble the Phases of the SHS into a single data set, while Python version 3.9.7 was used to script the LR, RF, and ANN models.

## Results

Numbers of SHS participants reporting hypertension, diabetes, and positivity for PAD are reported in Table 1.

More females than males participated in Phase V, comprising over 60% of the study participants. In addition, women reported higher percentages of hypertension, diabetes, and PAD than did men.

**FIGURE 2:**
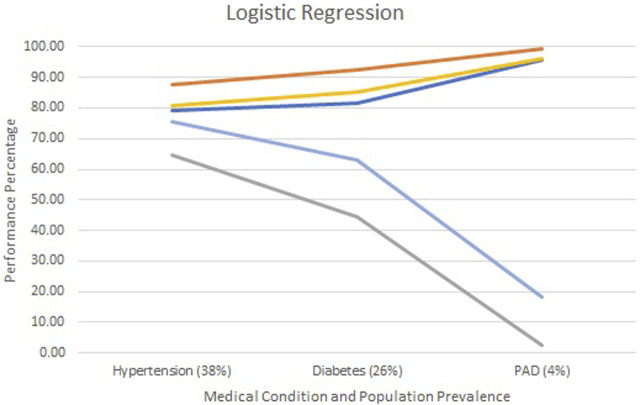
Screening test diagnostics for logistic regression. 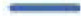
Accuracy 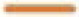
Specificity 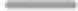
Sensitivity 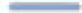
PPV 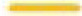
NPV.

**FIGURE 3:**
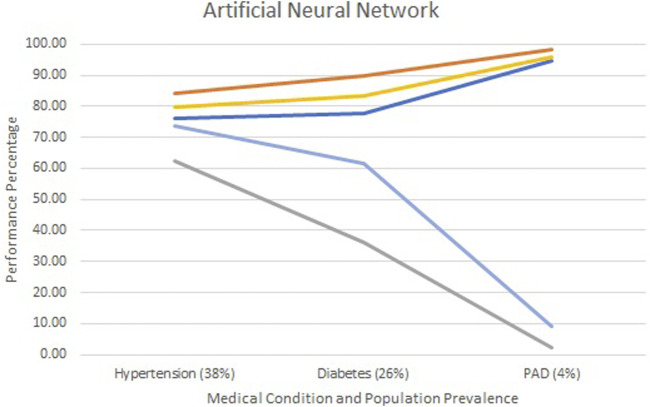
Screening test diagnostics for artificial neural networks. 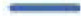
Accuracy 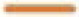
Specificity 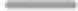
Sensitivity 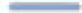
PPV 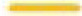
NPV.

**FIGURE 4:**
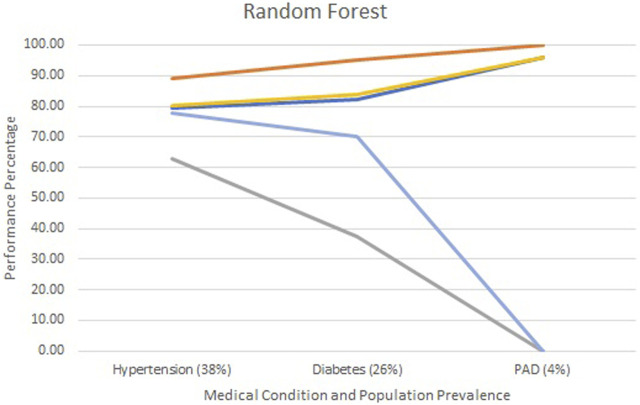
Screening test diagnostics for random forest. 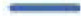
Accuracy 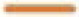
Specificity 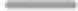
Sensitivity 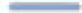
PPV 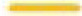
NPV.

Figures 2–4 show each model’s accuracy, sensitivity, specificity, NPV, and PPV and reflect similar performance patterns among all models. The PPV and sensitivity measures seem to suffer the most as the outcome prevalence declines, which is what is typically observed for a traditional laboratory-based screening test. PPV and sensitivity decline for all models but remain parallel for LR and appear to converge within the ANN and RF models. As PPV and sensitivity decline for the RF model, they converge to zero at an outcome prevalence of 4% (PAD). Specific numerical values for each model metric are recorded in Table 2 for all three chronic conditions.

**TABLE 2:** Summary of screening test diagnostics by method: logistic regression, artificial neural network, and random forest are represented by LR, ANN, and RF.

Metric	Model and chronic condition								
	Hypertension			Diabetes			PAD		
	LR	ANN	RF	LR	ANN	RF	LR	ANN	RF
Accuracy	78.99	76.00	79.35	81.49	77.66	82.18	95.57	94.56	95.99
Specificity	87.52	84.21	89.24	92.28	89.92	95.31	99.47	98.44	100.00
Sensitivity	64.79	62.41	62.88	44.59	36.06	37.26	2.40	2.07	0.00
PPV	75.61	73.62	77.75	62.88	61.77	70.13	18.06	9.04	0.00
NPV	80.63	79.87	80.12	85.08	83.52	83.88	96.06	96.00	95.99

All three models reported accuracy and specificity values that increased as the condition’s prevalence declined. These two measures are roughly 95% or higher for PAD, regardless of the model selected. Conversely, sensitivity and PPV decreased as the prevalence declined, largely due to the increased number of false positives. Although poor, the LR model reported the greatest PPV of 18% for PAD, as compared to the ANN and RF, which were at 9% and 0%, respectively.

The formulas for sensitivity and PPV in Figure 1 give insight to the effect of false and true positives on these two metrics. Traditional laboratory screening tests’ performance metrics are usually determined via a gold standard. As the prevalence of the condition declined, so did the number of true positives, while that of false positives increased, driving down both the sensitivity and PPV. Accuracy remained high as the true negatives grew, inflating these metrics.

## Discussion

LR, ANNs, and RFs are popular methods in the burgeoning world of AI and ML. Although these methods are quite different from one another, we can see that their performance metric trends are similar in screening for disease outcomes with varying prevalences. These performance metrics give the developers of these methods an idea of how a specific *in silico* screening method will perform in the population it was designed to serve based on the prevalence of the outcome.

Although these algorithms may have high predictive power, as measured in terms of predictive accuracy, some are criticized for lacking any causal reasoning [30]. For example, ANNs may give reliable predictions for the end users; however, these end users do not know how the algorithm came to a particular conclusion. Thus, they are “black boxes” contributing little to understanding a condition’s cause.

Regardless of the method used, the PPV declined in parallel with the overall prevalence of the condition. The type of *in silico* modeling approach is still subject to the same limitations as those of traditional lab-based screening tests, an important factor to remember as online screening tests become more widespread. This study reminds us that regardless of the approach used, *in silico* AI and ML screening tests are not “magic bullets.” Their performance is still limited by the prevalence of the disease in the populations they are intended to serve.

## Data Availability

Publicly available datasets were analyzed in this study. This data can be found here: https://strongheartstudy.org/.
